# Woody Plant Life-Form Structure Reflects Major Ecological Gradients Within a Protected Temperate Ecosystem from Romania

**DOI:** 10.3390/plants15081194

**Published:** 2026-04-13

**Authors:** Madalina Iordache, Catalina Marinescu, Mihai Valentin Herbei, Ioan Gaica, Daniel Dicu, Nicoleta Ianovici

**Affiliations:** 1 Department of Sustainable Development and Environmental Engineering, Faculty of Agriculture, University of Life Sciences “King Mihai I” of Timisoara, 300645 Timisoara, Romania; 2 Doctoral School of Plant and Animal Resources Engineering, University of Life Sciences “King Mihai I” of Timisoara, 300645 Timisoara, Romania; 3 Research Centre of Bioresources, Environment and Geospatial Data, University of Life Sciences “King Mihai I” of Timisoara, 300645 Timisoara, Romania; 4 Doctoral School, University of Petrosani, 332006 Petrosani, Romania; 5 Department of Biology, Faculty of Chemistry, Biology and Geography, West University of Timisoara, 300223 Timisoara, Romania; nicoleta.ianovici@e-uvt.ro; 6 Environmental Biology and Biomonitoring Research Center, West University of Timisoara, 300223 Timisoara, Romania

**Keywords:** woody flora, ecological indicators, Raunkiaer classification, Ellenberg indicator values, mesophilous species, mesothermal species, soil reaction, phytogeographical spectrum, floristic composition

## Abstract

This study, conducted in the Cheile Nerei–Beușnița National Park (southwestern Romania), tested the hypothesis that the life-form structure of woody plants reflects the main ecological gradients of moisture, temperature, and soil reaction in a temperate protected ecosystem characterised by sub-Mediterranean influences and a predominantly calcareous substrate. The analysis focused on the woody flora of the area, comprising 64 species belonging to 22 families, and included the assessment of life-form structure, phytogeographical spectrum, and ecological preferences based on Ellenberg indicator values. Life forms were classified according to Raunkiaer’s system, identifying megaphanerophytes, mesophanerophytes, and nanophanerophytes. The woody flora was dominated by nanophanerophytes, followed by megaphanerophytes and mesophanerophytes, indicating a complex vertical structure. The phytogeographical spectrum showed a predominance of European elements, alongside Eurasian and sub-Mediterranean components. Ecological analysis revealed a dominance of mesophilous and mesothermal species, consistent with mesic and temperate environmental conditions. Soil reaction preferences were mainly basiphilous and neutrophilous, reflecting the calcareous substrate, with vertical differentiation of ecological niches between tree and shrub layers. The high proportion of native species (>90%) and the limited presence of alien taxa indicate a high level of ecological integrity and resistance to biological invasions. Overall, the results demonstrate that the structure of woody plant life forms and their ecological preferences accurately reflect the main ecological gradients of the ecosystem. The combined use of life-form spectra and ecological indicator values provides a useful framework for assessing ecosystem structure, stability, and conservation status in temperate protected areas.

## 1. Introduction

Woody plants represent key structural components of terrestrial ecosystems, defining the vertical architecture of vegetation and regulating essential biotope factors and biogeochemical processes [1,2,3,4,5]. Owing to their longevity and spatial dominance, they integrate environmental conditions over extended temporal scales, indicating the cumulative influence of climatic, edaphic, and topographic factors [6,7]. Consequently, woody species play a decisive role in shaping ecosystem structure, regeneration patterns, and overall stability [8,9,10].

The distribution of woody species is closely linked to major abiotic factors, particularly water regime, temperature, and soil reaction, which control their presence and abundance and structure plant communities along ecological gradients [11,12,13]. In contrast to short-lived herbaceous species, woody plants respond primarily to stable environmental constraints, capturing prevailing ecological conditions while reducing sensitivity to short-term variability [14,15]. This characteristic makes them reliable indicators of baseline ecosystem conditions. Differences in adaptive strategies between woody and herbaceous species further reinforce this role, as woody plants tend to display more stable spatial patterns and a stronger response to environmental filtering processes [15,16,17].

Within this framework, the ecological preferences of woody species—such as hygrophilous, mesophilous, or xerophilous behaviour, together with their thermal and edaphic affinities—provide a basis for identifying environmental heterogeneity and interpreting the main ecological gradients of an ecosystem. The composition of woody flora, therefore, supports the understanding of transitions between hydric, thermal, and edaphic conditions and contributes to the assessment of plant community structure [18,19,20].

The analysis of woody flora is also essential for evaluating the conservation status of protected ecosystems and for supporting monitoring and adaptive management strategies. Previous studies have shown that the distribution and dynamics of woody populations are primarily controlled by edaphic conditions and site hydrology [21], while life-form analysis represents a useful tool for assessing ecosystem responses under both natural and disturbed conditions [22]. In addition, ecological indicator values associated with woody species can reveal deviations from expected ecological conditions, including processes related to biological invasions and ecosystem instability [23,24].

Recent studies further emphasise that vegetation structure and species composition reveal ecological trajectories shaped by environmental drivers, including climatic variability and edaphic constraints [9,15,25,26,27,28]. In this context, the structure of woody flora—particularly life-form composition and ecological preferences—can be used to infer ecological gradients and detect changes in ecosystem functioning. The integration of ecological traits into vegetation analysis thus provides a robust framework for understanding ecosystem stability and resilience under changing environmental conditions [29,30,31,32].

Woody communities in protected areas play a central role in ecosystem organisation, functioning, and stability [33,34,35]. Their persistence and structural importance confer particular value as indicators of habitat condition, ecological continuity, and anthropogenic influence [36,37]. The presence or absence of key woody species can indicate land-use history, disturbance regimes, and regeneration potential, while their ecological preferences help identify underlying environmental gradients [38,39]. In this context, the analysis of woody flora offers an integrative and consistent approach for identifying ecological gradients within protected ecosystems.

This study is based on the hypothesis that the distribution of woody plant life forms within the studied ecosystem is not random, but captures structured ecological gradients associated with moisture, temperature, and soil reaction. The underlying assumption is that the ecological structure of woody species, expressed through their life forms and ecological preferences, can be used to infer the main environmental gradients shaping the ecosystem.

Despite the long-standing use of life-form classifications and ecological indicator values in plant ecology, their combined application remains relevant for understanding the ecological organisation of plant communities, particularly within specific regional contexts [40,41,42]. Although comprehensive floristic surveys [42] that include all plant groups can provide detailed information about ecological gradients, such approaches are often labour-intensive and require extensive sampling and taxonomic effort. In contrast, woody vegetation, through its longevity and structural role, reflects the interaction between climatic, edaphic, and hydrological factors [43,44]. However, the extent to which the ecological structure of woody flora can be used to infer ecosystem-level gradients remains insufficiently explored in many protected areas of Central and Eastern Europe [43,44].

In this context, the present study aims to analyse the ecological structure of woody vegetation by integrating life-form classification with Ellenberg indicator values. Specifically, the study examined how woody plant life forms are distributed along ecological gradients related to moisture, temperature, and soil reaction, and evaluated whether these patterns reveal structured ecological gradients rather than random species assemblages.

The conceptual approach of the study is based on the assumption that Ellenberg indicator values describe species’ ecological preferences rather than direct environmental measurements. Therefore, this study tests whether, in the case of the analysed protected ecosystem, the ecological preferences of woody species can reliably describe the actual environmental conditions.

We hypothesise that the life-form structure of woody plants is significantly associated with the main ecological gradients of the ecosystem and that the distribution of species reflects a predominantly mesophilous and mesothermal ecological framework. This expectation is grounded in the temperate biogeographical and nemoral character of the study area, which is characterised by deciduous forest vegetation and generally moderate climatic conditions. In accordance with these conditions and the presence of calcareous substrates, most woody species are expected to exhibit mesophytic moisture preferences, mesothermal temperature requirements, and basiphilous or neutrophilous soil affinities. At the same time, secondary xeric, thermophilous, and edaphically differentiated elements are anticipated to occur as subordinate components shaped by local habitat heterogeneity, resulting in a structured but predominantly mesophilous–mesothermal ecological pattern.

The formulation of this working hypothesis was further motivated by recent studies [17] questioning the ability of ecological indicator values to consistently capture environmental conditions beyond broad macroclimatic patterns, highlighting their limited performance in capturing fine-scale variability. For example, in certain cases, it was evidenced that Ellenberg indicator values reliably confirm regional temperature differences but perform inconsistently at fine spatial scales within forests [17]. Against this background, the present study evaluates whether the life-form structure of woody plants, corroborated by biotope ecological preferences, can still provide a meaningful indicator of major ecological gradients within a temperate forest ecosystem.

The aim of this study was to assess whether the life-form structure of woody plants indicates major ecological gradients within a protected temperate ecosystem, as inferred from their ecological preferences for moisture, temperature, and soil reaction, expressed through Ellenberg indicator values. More specifically, the present study investigated the ecological structure of woody vegetation within the studied protected temperate ecosystem by integrating life-form classification and ecological indicator values. Specifically, the objectives were as follows: (1) to examine the relationship between woody plant life forms and ecological gradients (moisture, temperature, and soil reaction) using Ellenberg indicator values; (2) to evaluate whether the observed distribution patterns reflect structured ecological gradients or random species assemblages, thereby providing insight into the ecological organisation of the woody flora. Overall, this study evaluates whether the ecological structure of woody flora can serve as a reliable indicator of environmental gradients and ecosystem organisation, providing a basis for interpreting vegetation structure and ecological functioning in protected temperate ecosystems.

## 2. Materials and Methods

### 2.1. Site Description

**Figure 1:**
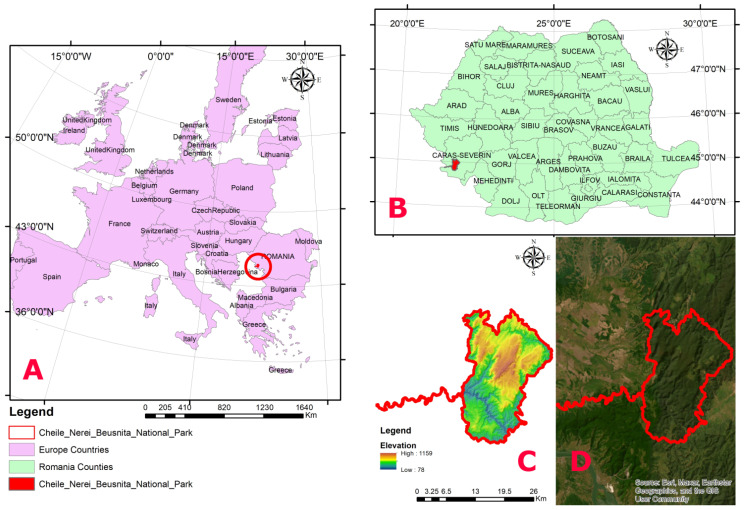
Cheile Nerei–Beușnița National Park: location within Romania (**A**) and within Europe (**B**); elevation map (**C**) and satellite map (**D**) [46].

Cheile Nerei–Beușnița National Park (44°56′21″ N, 21°51′24″ E) is located in southwestern Romania, within the Western Carpathians (Figure 1), and covers approximately 37,000 ha (36,660.65 ha), of which 8800 ha are designated as strictly protected reserves. The park was established in 1990 [45], although localised protection measures have been in place since 1943. Altitudes range up to a maximum of 1160 m, with a mean elevation of 600–800 m within the park. The entire park area falls within the continental bioregion. The bioclimatic belts are colline and montane, including the lower montane and middle montane sub-belts.

The dominant geological substrate consists of strongly tectonized and karstified Jurassic and Cretaceous limestones. The alternation of carbonate bands with impermeable rocks and their arrangement in narrow strips generate a distinctive, highly fragmented and morphologically diverse relief, as well as a disorganised hydrographic network. Slope exposures reflect the general NNE–SSW orientation of valleys and ridges, while in the southern half of the park, the Nera River valley, which crosses the area along a SE–NW direction, favours both southern and northern exposures.

The park’s geographical position places it within a temperate-continental climatic zone with Mediterranean influences: mean annual temperatures range between 6 and 11 °C (approximately 10–11 °C in low peripheral areas and around 6 °C at higher elevations), with mean January temperatures between −3 and −6 °C and mean July temperatures between 16 and 19 °C. Mean annual precipitation ranges from 700 to 1100 mm, with an approximately even distribution between the cold and warm semesters, a characteristic feature of southwestern Romania [46].

### 2.2. Plant Life-Form Structure

The life-form structure of the woody flora was analysed using Raunkiaer’s biological form classification [47]. Species were assigned to life-form categories based on the position of their perennial renewal organs relative to the soil surface, reflecting their ecological adaptation to environmental conditions. As the study focused exclusively on woody vegetation, the analysis was restricted to phanerophytic life forms, namely megaphanerophytes, mesophanerophytes, and nanophanerophytes. The classification of species into these categories was carried out according to the criteria defined in the Raunkiaer system as described in the Romanian phytogeographical literature [48,49]. The life-form spectrum was analysed quantitatively by determining the number of species and their relative frequency (%) within each life-form category.

### 2.3. Phytogeographical Spectrum

The assignment of species to phytogeographical categories (geoelement groups) was carried out according to Romanian phytogeographical literature [48,49]. The phytogeographical categories were analysed quantitatively by calculating the number of species and their relative proportion (%) within each category. The phytogeographical spectrum of the woody flora was established based on the native distribution ranges of plant species at continental and supra-regional scales, with emphasis on their primary centres of origin rather than their current distribution. Species were assigned to major phytogeographical categories according to the spatial coherence of their natural ranges and recognised centres of distribution. Species of non-native origin were treated separately, based on their biogeographical provenance and introduction history (native, allochthonous, or archaeophyte). Native species were defined as taxa occurring spontaneously within their natural range in Romania, without direct human intervention. Alien species were considered taxa introduced intentionally or accidentally outside their natural range. Archaeophytes were treated as a distinct category of alien species, representing long-established taxa that are well naturalised and frequently encountered in the wild, and exhibit stable, non-invasive behaviour in Romanian ecosystems; they are no longer regarded as invasive [50,51].

### 2.4. Ecological Preferences for Moisture, Temperature, and Soil Reaction

The ecological requirements of the investigated plant species were evaluated with respect to soil moisture, temperature regime, and soil reaction (pH) using Ellenberg’s ecological indicator values [52]. For each species, an ecological preference category was assigned and interpreted qualitatively, reflecting its affinity or tolerance to the respective environmental factors. Soil moisture preferences were expressed as: amphitolerant (euryhydric), xerophyte, xeromesophyte, mesophyte, mesohygrophyte, hygrophyte, and hydrophyte. Temperature preferences were classified into: amphitolerant (eurythermal), cryophile, microthermal, mesothermal, moderately thermophilic, and thermophilic. Soil reaction preferences were assigned to: amphitolerant (euryionic), strongly acidophilous, acidophilous, acido-neutrophilous, slightly acido-neutrophilous, neutrophilous, neutro-basophilous, and basophilous categories. All classifications and ecological attributes were assigned according to the criteria and statistical frameworks provided by Romanian phytosociological literature [48,49]. Principal Component Analysis (PCA) was conducted using PAST 3.x (PAleontological STatistics) to identify the principal ecological gradients associated with plant life forms, based on variables reflecting the numerical distribution of species according to their requirements for moisture, temperature, and soil reaction considered at the level of each plant life-form (megaphanerophytes, mesophanerophytes, and nanophanerophytes).

### 2.5. Selection of Plant Life Forms and Ecological Variables

Despite the recognised ecological importance of woody vegetation, the relationship between woody life-form structure (megaphanerophytes, mesophanerophytes, and nanophanerophytes) and environmental preferences remains insufficiently explored, particularly in protected temperate ecosystems characterised by heterogeneous microhabitats. It is still unclear to what extent the distribution of woody life forms is structured by moisture, thermal, and edaphic requirements, and whether these gradients can be reliably inferred from woody species composition alone.

The analysis of woody vegetation life forms represents a relevant functional approach for investigating ecological gradients, as previous studies conducted in protected areas have demonstrated that life-form structure provides essential information on regeneration patterns and ecosystem resilience, highlighting their capacity to respond to environmental conditions and long-term disturbances [53]. Studies carried out in protected areas with contrasting ecological conditions show that ecosystem structure and diversity are closely linked to life-form composition. Analyses of flora conducted in such areas indicate that the dominance of trees and shrubs provides a stable structural framework, even under conditions of anthropogenic pressure and stress [54]. Thus, the use of structural indicators and life-form spectra allows the definition of a reference state for conservation assessment and for monitoring endemic or protected species.

In some cases, life-form type may reflect ecosystem dynamics more accurately than species diversity alone [22,26]. It has also been shown that trees can effectively describe the structure and identity of plant communities when analysed from the perspective of life forms and their ecological preferences toward biotope factors, functioning as integrators of environmental conditions [18]. In this context, life-form analysis has proven to be an essential functional tool in both natural ecosystems and those affected by disturbances, allowing the assessment of the risk that certain natural or managed interventions may compromise tree regeneration or the continuity of the woody layer [22]. Therefore, life forms, together with their associated ecological preferences, can be used as indicators of ecosystem integrity. The importance of this approach is further supported by studies showing that different plant life forms exhibit distinct responses to environmental factors and landscape structure, and that their distribution reflects both dominant ecological conditions and the degree of disturbance [26]. Thus, the functional approach based on life forms provides an appropriate framework for interpreting the organisation, stability, and long-term functioning of ecosystems.

Ecological gradients are fundamental drivers of plant community composition and structure. Among the most widely used tools for detecting such gradients are Ellenberg indicator values (EIV), which quantify species’ ecological preferences for temperature, moisture, soil reaction, light, and nutrients. By integrating these values at the species or community level, it becomes possible to infer environmental conditions even in the absence of long-term instrumental measurements. Recent studies have demonstrated that Ellenberg-based analyses can reveal directional vegetation changes associated with climate shifts, habitat fragmentation, hydrological alteration, and successional dynamics. However, most studies have focused on herbaceous communities, while fewer have addressed the specific role of woody species as integrators of ecological gradients.

The application of Ellenberg indicator values (EIVs) in the analysis of woody flora in Romania is supported both by their long-standing use in European vegetation ecology and by recent methodological developments that have expanded their applicability in Southeast Europe [43,44]. The use of woody species in ecological analyses offers the advantage of a solid ecological documentation base for most taxa, for which well-established Ellenberg indicator values are available. Previous studies have shown that changes in Ellenberg values can be interpreted as responses to climatic and edaphic gradients even in the absence of complete land-use data series [25]. The integration of these values allows the quantification of ecological preferences for temperature, moisture, and soil reaction and facilitates the inference of environmental conditions in ecosystems where long-term instrumental data are limited. By analysing ecological preferences for biotope factors, woody species can be readily integrated into comparative analyses, conceptual models, and monitoring studies, thereby increasing the comparability and reproducibility of results.

Although indicator values may be incomplete or insufficiently documented for some regional species, recent studies have demonstrated that robust statistical methods allow the estimation of missing values with a high level of accuracy and ecological consistency [43]. Community-weighted Ellenberg indicator values represent an effective tool for detecting ecological changes and gradients, even in situations where direct environmental data are incomplete or variable [25].

Some studies have shown that the Ellenberg index has a “good” to “excellent” capacity to predict the distribution of certain woody species, confirming the usefulness of EIVs in modelling species distribution and highlighting the predictive value of ecological traits in the context of climate change. These findings suggest that woody species reflect well-defined climatic limits, and that shifts in these limits may lead to the restructuring of forest ecosystems [55]. Within the biogeographical context of Romania, located at the intersection of Central European, Balkan, and sub-Mediterranean elements, the use of Ellenberg indicator values is particularly relevant for detecting fine ecological gradients. In this framework, the application of EIVs represents not only a descriptive approach, but also an effective analytical tool for interpreting ecological gradients and for supporting conclusions regarding the organisation and functioning of the studied ecosystem [43].

In conclusion, the use of woody vegetation life forms, in correlation with Ellenberg indicator values, provides a coherent and functional methodological framework for the analysis of ecological gradients in the studied temperate protected ecosystem.

### 2.6. Methodology Limitations

The classification of species into life forms (megaphanerophytes, mesophanerophytes, and nanophanerophytes) was conducted in this study at the level of species composition, with the objective of characterising the functional and ecological spectrum of the woody flora, rather than analysing population structure or quantitative dominance. This approach was adopted because life form represents a stable functional trait of a species and is not conditioned by the numerical abundance of individuals at the local scale; consequently, the life-form spectrum reflects ecological strategies and environmental conditions, rather than population structure. The abundance of individuals may reflect recent numerical fluctuations or local disturbances, whereas the presence of woody species and their affiliation with specific life forms integrates dominant ecological conditions over the long term. For this reason, the analysis of the studied protected ecosystem focused on the species level rather than the population level. It is possible that, within the analysed ecosystem, some species are represented by a higher number of individuals than others; however, this aspect does not influence the interpretation of the life-form spectrum, as the analysis targets the distribution of functional strategies at the species level, not quantitative dominance. Given that the objective of the study was to evaluate the relationship between species’ ecological requirements (according to Ellenberg indices for moisture, temperature, and soil reaction) and the functional structure of woody flora, we consider that the use of a species-level life-form spectrum is more relevant than weighting individuals numerically.

Our methodological approach is consistent with studies demonstrating that life form is a major determinant of the distribution and abundance of woody species, whereas the local abundance of individuals may vary independently of a species’ ecological requirements. For example, megaphanerophytes may be locally less abundant (few individuals) but more frequent at the landscape scale (occurring in multiple plots), whereas nanophanerophytes may be locally abundant but rarer at broader spatial scales [56]. Therefore, local numerical abundance represents a different indicator than ecological importance or representativeness. Consequently, frequency (presence–absence) patterns are ecologically more robust and more strongly correlated with phylogeny and life form than patterns based strictly on individual counts. In other words, not all ecologically important species are numerically abundant. Other studies have shown that the regional distribution of woody species is primarily determined by functional traits such as life form, dispersal strategy, and longevity, rather than by local numerical abundance [57]. In addition, some studies have demonstrated that Ellenberg indicators are more sensitive and “faster” in lower vegetation layers, and that the species–environment relationship weakens from lower to upper strata: it is clearer in the lower layers and more diffuse in the tree layer, because in woody species the signal is temporally filtered [58], which explains the stability provided by the characterisation of persistent woody life forms. Thus, the analysis adopted in the present study, based on species presence and their affiliation with life forms, is appropriate for interpreting ecological gradients reflected by Ellenberg indicator values.

Future analyses may integrate quantitative data on individual abundance to explore relationships between life forms and population structure; however, this approach is beyond the scope of the present study.

## 3. Results

The woody flora of the studied protected area comprises 64 species belonging to 22 plant families (Table 1).

**Figure 2:**
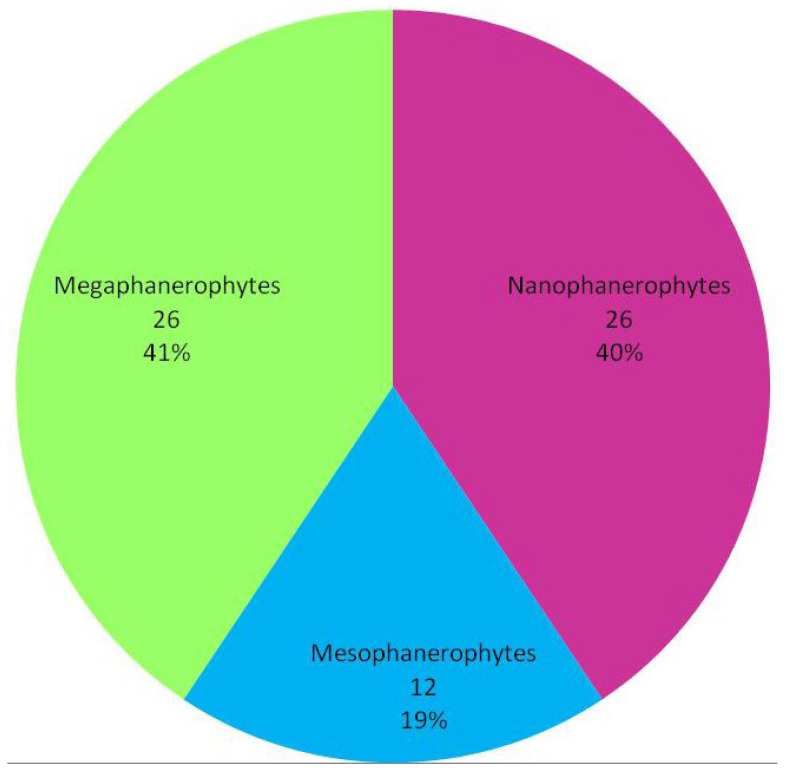
Numerical distribution of plant life-forms (nano-, meso-, and megaphanerophytes) within the studied woody flora.

The results of the analysis showed that the woody flora of the studied protected area is dominated by nanophanerophytes (26 species), followed by megaphanerophytes (26 species) and mesophanerophytes (12 species) (Figure 2).

**Figure 3:**
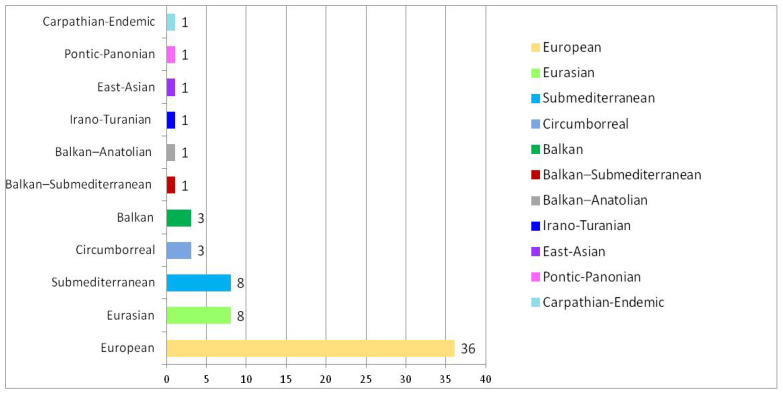
Distribution of the phytogeographical elements within the studied woody flora.

The phytogeographical spectrum of the woody flora in the studied protected park comprises 11 phytogeographical types (Figure 3), with species of European origin being the most numerous. However, the woody flora comprises a mixture of European, Eurasian and sub-Mediterranean dominant elements, suggesting the overlapping of ecological influences elements and the convergence of species adaptation processes over time.

### 3.1. Analysis of Woody Flora According to Ecological Preferences for Moisture

**Figure 4:**
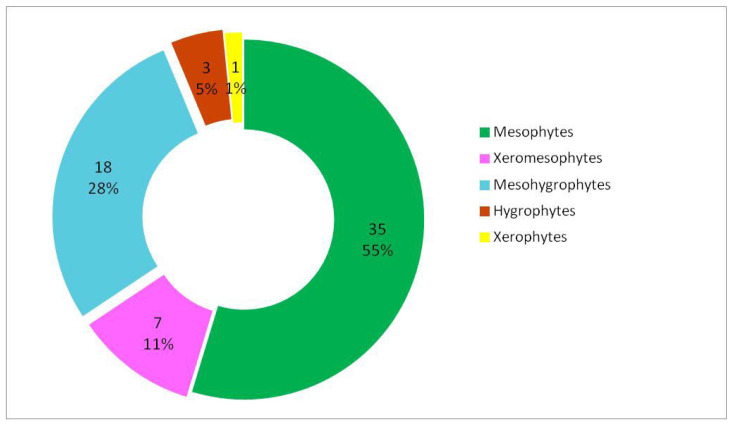
Distribution of soil moisture preferences within the studied woody flora.

**Figure 5:**
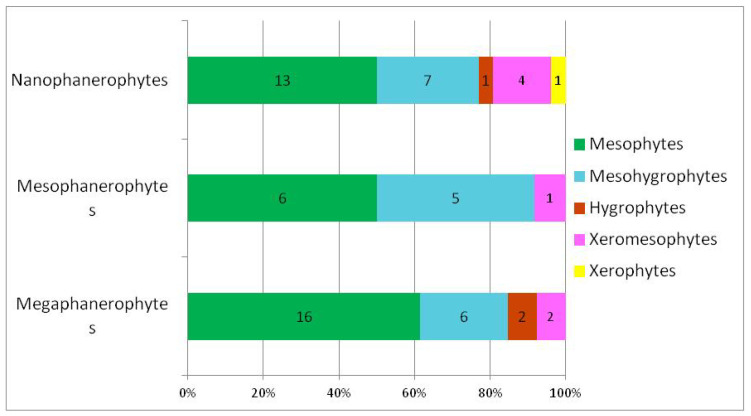
Distribution of ecological preferences for moisture across phanerophyte life-form categories.

The distribution of woody species according to their moisture requirements (Table 2) indicates a clear predominance of mesophytes, which comprise 35 species (approximately 55%) of the total analysed (Figure 4), which indicate a moderate hydric regime. Among these, 16 are megaphanerophytes, 5 mesophanerophytes, and 14 nanophanerophytes, indicating a balanced participation of trees and shrubs within the mesic segment of the hydric gradient (Figure 5).

### 3.2. Analysis of Woody Flora According to Ecological Preferences for Temperature

**Figure 6:**
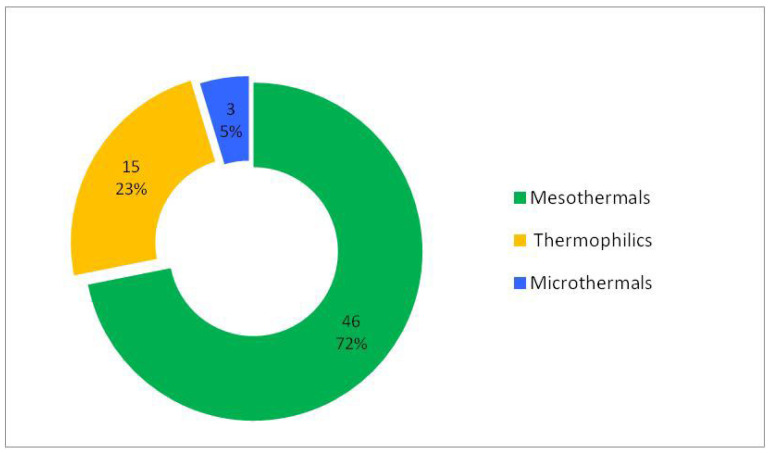
Distribution of soil temperature preferences within the studied woody flora.

**Figure 7:**
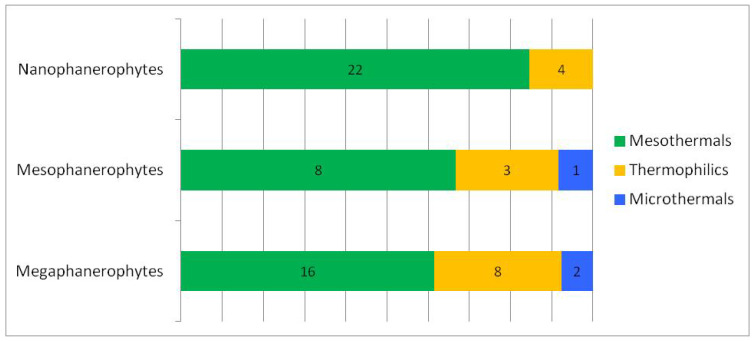
Distribution of ecological preferences for temperature across phanerophyte life-form categories.

Thermally, the woody flora of the studied ecosystem is dominated by mesothermal species (approximately 72%) (Table 2, Figure 6), which indicates a moderate temperate climatic character of the ecosystem, while thermophilous species form a secondary but well-defined component (approximately 23%), confirming sub-Mediterranean influences or locally warmer conditions. Microthermic elements are scarce (approximately 5%) and are represented by tree species (mega- and mesophanerophytes) (Figure 7), indicating the presence of restricted cooler microhabitats. This structure reflects a temperate climatic framework with localised warm influences.

### 3.3. Analysis of Woody Flora According to Ecological Preferences for Soil Reaction (pH)

**Figure 8:**
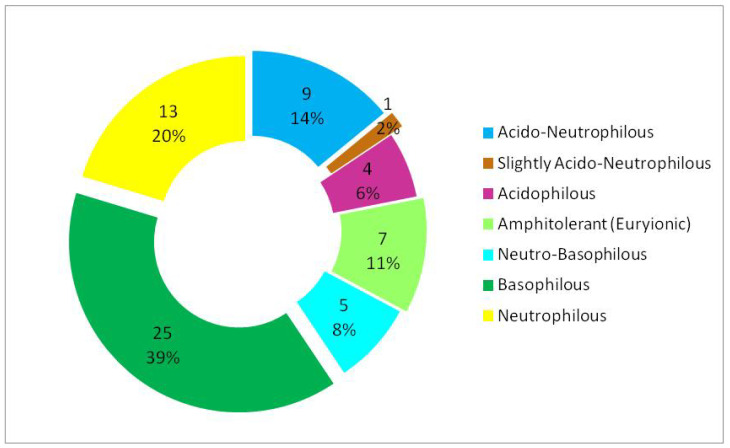
Distribution of studied woody species in relation to soil reaction (pH).

The woody flora present in the studied ecosystem is dominated by basiphilous species (approximately 39%) (Table 2, Figure 8). The neutrophilous segment is also well represented (approximately 20%), indicating a significant influence of the calcareous substrate or of the dominant neutral to basic soils [46] within the ecosystem. Acidophilous species have a low proportion (approximately 6%), indicating a limited presence of acidic substrates (Figure 8).

**Figure 9:**
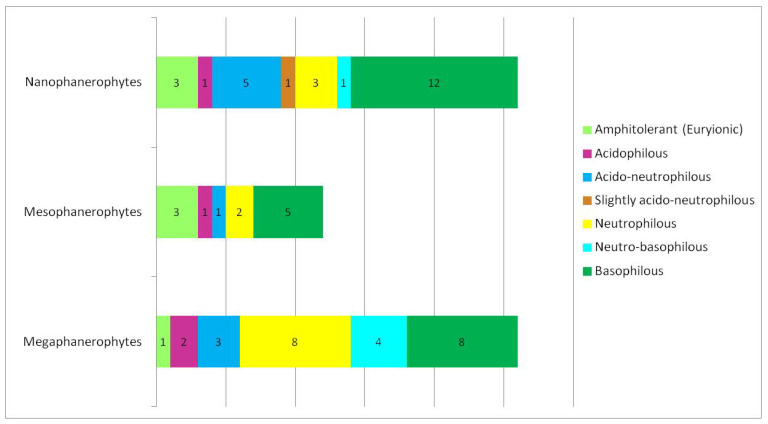
Distribution of ecological preferences for soil reaction (pH) across phanerophyte life-form categories.

Overall, the structure of soil reaction in relation to life forms (Figure 9) suggests an ecosystem with a predominantly neutral–basic tendency, characterised by a moderate heterogeneity of soil pH.

## 4. Discussion

The species richness (64 plant species) of the studied ecosystem (Table 1) is comparable to that reported in other ecosystems, where a similar number of woody taxa (trees and shrubs) has been documented [59]. Considering the extent of the study area (approximately 37,000 ha; 370 km^2^), the recorded assemblage including mega-, meso- and nanophanerophytes, reflects a structurally diverse woody component. This number represents roughly 20–21% of the more than 300 woody species of forestry interest reported at the national level [60] for Romania. Such a proportion is not negligible, particularly considering that the national assessment includes all woody taxa occurring across the full range of Romania’s biogeographical regions and ecological contexts, encompassing alpine, steppe, sub-Mediterranean, Pontic and other specialised habitats, as well as rare, endemic, and relict species. In contrast, the present study captures a coherent ecological subset of the national woody flora, filtered by a temperate climatic framework, local edaphic conditions, and the specific land-use and protection history of the area. The recorded woody flora is dominated by ecologically relevant mega-, meso- and nanophanerophytes, which are characteristic of temperate ecosystems and responsive to long-term environmental constraints. Consequently, the occurrence of approximately one fifth of the national woody flora within a single protected area reflects ecological representativeness rather than an attempt to achieve floristic exhaustiveness at the national scale. When related to surface area, the recorded richness corresponds to 64 woody species across 370 km^2^, or approximately 17.3 species per 100 km^2^ (equivalently per 10,000 ha). Although species density expressed per unit area is not a standardised metric for woody plants and does not scale linearly with area, this value provides a useful order-of-magnitude indication that the observed richness is substantial for a large protected landscape. From an ecological perspective, the presence of 64 woody species within a 37,000 ha area suggests pronounced habitat heterogeneity. The coexistence of diverse woody taxa likely reflects the combined influence of hydric, edaphic, and topographic gradients, as well as the presence of a mosaic of vegetation types, including forest stands, forest edges, shrub communities, and riparian habitats. Overall, the identification of 64 woody species, representing approximately one fifth of the woody flora reported at the national level, highlights the high ecological representativeness of the studied protected area. This finding supports the interpretation that the life-form structure of woody plants effectively reflects structured hydric, thermal, and edaphic gradients within a temperate ecosystem and confirms the interpretative value of woody life forms in the analysis of ecosystem structure and functioning.

The family Rosaceae is the numerically most represented, with 16 species, and has also been identified as the most species-rich family in other studies on woody plant biodiversity in temperate ecosystems [61], including forest ecosystems [62,63], urban arboretums [64], urban gardens [65], protected areas [66,67], and even cemeteries [68]. Lakicevic et al. (2022) [69] highlighted that the dominance of the Rosaceae family represents a typical characteristic of the dendroflora from temperate climatic regions, to which Romania also belongs and was explained through the excellent versatility of the species from this family when related to environmental conditions. Compared to other families, Rosaceae benefits from efficient dispersal strategies and high adaptability to various ecological conditions [62]. Thus, some woody species from the Rosaceae family have proven to be true ecosystem engineers [70], since they not only establish in the ecosystem in which they have arrived, but actively modify its biotope by altering the access to certain ecological factors (such as light) or by changing the structure and chemistry of the soil, thus having a profoundly transformative role. For example, it has been observed that the diversity of the Rosaceae family is important for the accumulation of organic carbon in the soil [71]. Such species capable of significantly impacting abiotic conditions determine effects dependent on the ecosystem context, producing changes that lead to the reduction in functional diversity (richness and dispersion) and to the homogenization of communities, through a strong habitat filtering that eliminates species sensitive to the abiotic factors captured by them and favours stress-tolerant taxa. In certain situations, some woody Rosaceae species conquer ecosystems becoming dominant due to particularities that make them capable of modifying the functional traits of other species (decrease in SLA, increase in dry matter content and seed mass); however studies have shown that there are also limits, critical thresholds (for example, reaching approximately 50% cover) beyond which the relationships between habitat and the diversity of these species collapse [70]. Consequently, studies show that the Rosaceae family contains woody species that can transform in the long term the structure of a forest ecosystem and regeneration processes, leading to the emergence of new, stable ecosystem states, which is why early detection and management are essential for preventing imbalances [70]. Other studies [72] have shown that the success of some woody Rosaceae species was determined by a combination of factors such as introduction by humans (including through cultural or religious exchanges), high competitiveness for resources and similarity to the native habitat, which facilitates their establishment and spread; in addition, a success factor can also be the taxonomic proximity of alien species to the local flora, which makes them adapt more easily and gives them greater chances to invade and dominate communities, thus explaining why some biological invasions are more successful than others. Also, in support of these statements, there are studies [73] which show that the success of the Rosaceae family has a historically attested antiquity. A comparison of palynological (73 spectra) and anthracological (9 sites, 305 samples) during the Neolithic period (5200–2200 BC) in the southern Scheldt basin (western Europe) provided evidence that the Rosaceae family fulfilled a clear role already in the Neolithic landscape, being characteristic of forest edge areas and reflecting ecotone vegetation, that is the transition between forest and open spaces; species from this family were then associated with edge forests and more open and light-rich areas, where they appeared alongside shrubs that preferred light or semi-shade and were typical for intermediate successional stages; in this context, their presence suggests that the vegetation was not a compact forest one, but also included fragmented or open areas, resulting either from the natural dynamics of vegetation, or from human activities such as deforestation, wood use or habitation; in conclusion, the Rosaceae family functioned then as an indicator of forest edges and mosaic landscapes, highlighting for the present the existence of open or semi-open habitats in the Neolithic period, possibly influenced by human activity [73].

Of the total number of woody species, only four are non-native (i.e., not naturally occurring within the studied region) (Table 1): *Juglans regia* (megaphanerophyte; native to Southwest and Central Asia) and *Morus alba* (megaphanerophyte; native to East Asia)—which are considered archaeophytes (i.e., a distinct category of allochthonous species introduced in historical times but currently naturalised), *Syringa vulgaris* (mesophanerophyte; native to Balkan Peninsula) and *Ulmus procera* (megaphanerophyte; probably originating from southern Europe and widely distributed through long-term human cultivation)—which are regarded as allochthonous species introduced outside their native distribution range.

The overwhelming dominance of native woody species (60 species, over 90%) suggests that the analysed ecosystem has not been profoundly disturbed by artificial introductions or biological invasions and that it preserves natural ecological processes of regeneration and competition. It appears that alien woody species establish with difficulty and encounter greater resistance within the woody flora, which makes woody species key pillars of invasion resistance, at least at the level of the woody layer, thereby underscoring their major importance as indicators of ecological continuity. In the case of the present study, we consider that the very low proportion of alien species indicates the presence of an effective ecological filter rather than a lack or failure of reproductive opportunities. Habitats in which woody ecological niches are already occupied by locally adapted native species become more difficult to penetrate. This interpretation is supported by other studies on spontaneous flora in Eastern Europe [74], which highlight that invasion processes are dominated mainly by alien woody species, representing the majority of naturalised and invasive taxa, including within protected areas. The prevalence of trees and shrubs in these processes emphasises the role of woody life forms as major agents of ecosystem restructuring. Other studies from Europe addressing the capacity of native tree species to establish during ecological succession in previously unoccupied biotopes [75] have shown that spontaneous development toward native forest is a long-term and slow process, often species-poor in large trees and dominated by the understory. Environmental factors (altitude, moisture, temperature) and herbivore pressure strongly control regeneration success, indicating that the biotope ecological filter regulates colonisation success. In this context, the results of the present study—indicating the dominance in the woody flora of the studied ecosystem of native megaphanerophyte and nanophanerophyte species with a specific ecological spectrum of biotope preferences—suggest the existence of a stable framework provided by these species, which have passed the long-term test of the biotope filter. These species are capable of limiting the establishment and expansion of invasive species and of expressing, through the existing life-form spectrum and associated ecological requirements, the dominant ecological gradient of the site. These conclusions further strengthen the rationale for the protection of the studied ecosystem and the continuity of its protected-area status.

The results of the analysis showed that mega- and nanophanerophytes clearly surpass mesophanerophytes, a pattern that has also been reported in other ecosystems [53,76] and represents an important ratio in describing ecosystem development. Results obtained in various protected areas indicate that the structure and composition of vegetation life forms determine the capacity of ecosystems to respond to disturbances. A study addressing the importance of natural regeneration of woody species in maintaining ecosystem diversity and functioning showed that the dominance of regeneration from seedlings relative to sprouts indicates high early recruitment, but also strong ecological selection at later stages, a process influenced by both environmental conditions and anthropogenic pressures [53]. Other studies have shown that the predominance of megaphanerophytes and nanophanerophytes confirms the dominant role of trees, associated with deeper soils and more favourable moisture and temperature conditions [76].

These observations suggest that life-form type may, in some cases, be more important than total species richness, and that analysing woody vegetation structure according to this criterion allows the identification of regeneration strategies and ecosystem resilience potential.

The dominance of native megaphanerophyte and nanophanerophyte species in the woody flora of the studied ecosystem suggests the existence of a stable framework provided by these species, capable of limiting the establishment and expansion of invasive species.

The spectrum of the phytogeographical elements within the studied woody flora indicated a mixture of European, Eurasian and sub-Mediterranean dominant elements. This pattern of mixed phytogeographical elements within an ecosystem, as well as their dominance by one or a few numerically representative groups, is a natural phenomenon also reported in other scientific sources, which confirm that the flora of an ecosystem is not the result of a single origin but rather of the superposition of multiple phytogeographical sources [77].

The predominance of European elements, together with the significant presence of Eurasian and sub-Mediterranean elements in the woody flora of the studied ecosystem, indicates a complex phytogeographical structure, pointing to a biogeographical contact zone where multiple floristic influences intersect. This combination suggests that the woody flora of the protected park cannot be interpreted as deriving from a single floristic source. Instead, the observed phytogeographical structure is the result of long-term historical and ecological processes, including successive species migrations, regional climatic variations, and selection driven by local environmental conditions. In this context, the convergence of adaptive processes has enabled the coexistence of species with different phytogeographical origins but compatible ecological requirements, leading to the assembly of a functionally coherent woody flora. This situation is consistent with contemporary biogeographical models, which emphasise the dynamic role of source pools and local ecological filters in shaping plant communities, particularly in bioclimatic transition zones [77]. Thus, the studied woody flora can be interpreted as the outcome of an interaction between biogeographical legacy and long-term ecological adaptation processes, rather than as a simple product of the geographical origin of its constituent species. The observed phytogeographical spectrum does not contradict but rather complements models proposed in the scientific literature [78], indicating that the current structure of woody flora results from the interaction between historical biogeographical filters and present-day ecological conditions, neither of which alone is sufficient to explain the identified diversity and distribution patterns.

### 4.1. Woody Flora and Moisture Preferences

The dominant presence of mesophytes species within the studied ecosystem represents a highly important ecosystem indicator, as temporal analyses of the maintenance or loss of this dominance can provide insights into the evolutionary stage of the ecosystem. Some studies have shown that strictly mesophilous woody species dependent on fresh and moist soils exhibit a clear decline under conditions of pronounced water deficit and increasing temperatures, whereas species with a broader ecological amplitude or with thermophilous requirements display increased vitality and expansion tendencies [21]. These results confirm that woody flora accurately reflects dominant ecological gradients, particularly along the soil moisture axis, and that the structure of plant communities reorganises in response to hydric changes, even in the absence of direct anthropogenic interventions.

The mesohygrophytes represent a significant proportion, comprising 18 species (approximately 28%), suggesting stronger edaphic or microclimatic moisture influences in certain sectors of the studied area. The mesohygrophytes are evenly represented across the life-form spectrum: megaphanerophytes—6 species, mesophanerophytes—5 species, and nanophanerophytes—7 species (Figure 5). The xeromesophytes have a low representation (7 species), being mainly represented by nanophanerophytes, while the xerophytes species are isolated (one nanophanerophyte species), indicating a minor contribution of more exposed or drier habitats within the studied ecosystem.

Overall, the life-form structure correlated with hydric requirements in the studied ecosystem reflects a predominantly mesic ecosystem through the presence of mesophytes and mesohygrophytes, with a moderate heterogeneity of the moisture regime, in which both megaphanerophytes and nanophanerophytes contribute to the expression of the ecological gradient. Although numerically less represented, hygrophytes and xeromesophytes reflect the habitat types within the studied protected area, and their contribution is highly important, as studies have demonstrated that ecological species groups and taxa with a narrow ecological amplitude have indicator value in ecosystems by faithfully reflecting habitat conditions and the temporal evolution of ecological gradients [79]. The positioning of these species along the main ecological gradients, their decline over time, or even their disappearance from an ecosystem show that the analysis of vegetation requirements for environmental factors represents a universal language for indicating the evolution of plant communities and habitat conditions. Thus, even when the coenoflora of a grassland in the Kazakh Altai [79] was analysed through detailed floristic analyses integrating coenofloristic structure, ecological groups, and species environmental requirements, it was shown that this synthetic analytical method represents an essential tool for assessing ecosystem condition and conservation status, demonstrating the capacity of plant communities to simultaneously integrate signals of natural factors and anthropogenic disturbances.

The reduced presence of stenohydric species in the analysed ecosystem suggests that no recent or abrupt hydric changes that are also stable over time—such as waterlogging, pronounced drying, or strong fluctuations in water level—have occurred. Stenohydric species respond rapidly to such changes, and their absence or rarity indicates that the moisture regime has remained relatively constant over the medium and long term. This pattern is characteristic of mature and well-structured ecosystems, where long-lived woody species contribute to microclimatic regulation and to the buffering of hydric variability, thereby limiting the development of conditions favourable to strictly hygrophilous or strictly xerophilous taxa. Changes in the moisture regime represent a determining ecological factor in vegetation restructuring. An increase in soil moisture within an ecosystem leading to the onset of partial waterlogging processes has been shown to result in significant changes in phytocoenosis structure, manifested by the appearance of hygrophilous species, an increase in the proportion of mesohygrophytes, and the degradation of the tree layer, as demonstrated by one study [80]. This example shows that vegetation responds primarily to hydric changes, and that the distribution of ecological groups constitutes a sensitive indicator of environmental transformations, confirming the relevance of using species ecological requirements in the analysis of ecosystem functioning.

In conclusion, in the analysed ecosystem, moisture requirements indicate a predominance of mesophilous species, accompanied by a substantial proportion of meso-hygrophilous elements and a limited xeric component. This pattern suggests a predominantly mesic ecosystem with moderate hydric heterogeneity. Mesophilous species are almost equally represented by megaphanerophytes and nanophanerophytes, highlighting the structural complexity of the vegetation and the contribution of both tree and shrub layers to the expression of the hydric gradient.

### 4.2. Woody Flora and Temperature Preferences

The correlation between ecological preferences for temperature and life-form types shows that nanophanerophytes dominate within the mesothermal segment, megaphanerophytes have a significant proportion in both the mesothermal and thermophilous segments, while microthermy is almost exclusively characteristic of tree species. Overall, the observed structure describes a predominantly mesothermal ecosystem, with secondary thermophilous influences and a reduced microthermal segment, as expected from the climatic–edaphic description of the studied site. A clear dominance is exhibited by nanophanerophytes, followed by megaphanerophytes (Figure 7).

Most of the mesothermal species identified in our study are nanophanerophytes, representing the lower layer of woody vegetation. Although ecological indicator values (EIVs) are frequently used to highlight changes in plant community composition associated with climate warming, some studies on forest ecosystems have shown that understory plant communities do not always reliably indicate increases in regional temperatures, thereby accumulating a so-called “climatic debt” [81]. This thermal lag can be explained, at least in part, by the microclimatic buffering effect of forests, which creates a cooler and more stable environment beneath the canopy compared to the regional climate. In contrast to studies focusing on understory plant assemblages co-occurring within individual forest stands, the present analysis does not address fine-scale species associations or microclimatic differentiation within forest patches. Instead, woody species were considered based on their presence across the protected ecosystem as a whole, allowing ecological indicator values to be interpreted as proxies for broad, ecosystem-level conditions rather than for local microhabitat variability. Within this framework, EIVs function as general macroecological references, capturing dominant climatic and thermal tendencies of the ecosystem, which may explain their consistent performance in reflecting major ecological gradients.

Thus, the results obtained in our study are consistent with those of other studies demonstrating that ecological indicator values reliably reflect major temperature differences among ecosystems corresponding to the regional macroclimate, thereby emphasising that EIVs are not erroneous tools but effective large-scale indicators [82], even though their applicability may be limited when used to describe microclimate, fine environmental heterogeneity, and local ecological processes [81]. Some studies have shown that ecological variability must be analysed in relation to the ecosystem mean and ecological context rather than as an independent property [83], because woody plant traits tend to be more homogeneous (clustered) under specific latitudinal and climatic conditions, with temperature and latitude acting as the key factors controlling this variability. Temperature has been identified as a factor structuring woody vegetation over the long term even in humid ecosystems traditionally considered to be predominantly controlled by hydrological factors, indicating that climatic gradients and topography may exert a stronger influence than hydrology [84]. Other studies have also shown that temperature and moisture are the most important ecological factors for differentiating habitat types and are closely correlated with the degree of naturalness [59,82,85]. Furthermore, studies on native woody vegetation in ecosystems have demonstrated that the most important gradients structuring woody vegetation types are related to altitude, temperature, and precipitation regime [86]. The integration of regional spatial and temporal variation in temperature into woody vegetation structure has been well illustrated by a study showing that the number of woody plant families tracked temperature in a predictable manner both spatially and temporally throughout nearly the entire period since the last glaciation (approximately the last 14,000 years), with only minor delays during periods of rapid cooling. This finding indicates that present-day climate primarily controls species richness, while the effects of glacial climate are limited [87]. In the context of the arguments presented above, the analysis of woody species’ preferences for temperature and moisture in the studied ecosystem shows that these preferences are consistent with the biotope characteristics of the ecosystem as described in terms of climate and edaphic substrate. Ecological indicator values for temperature (EIVs) and the structure of the phytogeographical spectrum in the studied ecosystem therefore capture the dominant climatic tendencies of the ecosystem and can be interpreted as relevant macroecological references for the analysed ecosystem. This is because, as an expression of environmental conditions, certain evolutionary groups can persist and dominate, thereby determining the structure of species coexisting within a community [88].

### 4.3. Woody Flora and Soil Reaction (pH) Preferences

The woody flora present in the studied ecosystem is dominated by basiphilous species. The basiphilous spectrum is dominated by nanophanerophytes, whereas the neutrophilous spectrum is represented mainly by megaphanerophytes (Figure 9). This indicates that, among the three woody life-form types, the extremes of the vertical structure of the ecosystem are also those that define its overall gradient, namely nanophanerophytes and megaphanerophytes. It is likely that these life forms reflect the soil pH profile of the analysed ecosystem more faithfully and over shorter temporal intervals, given that some studies [58,89] have shown that the lower layers of woody vegetation respond more rapidly to variations in soil pH compared to larger-sized species in the upper canopy layer. Some studies on forest plantations have shown that the structure of woody communities during subsequent ecological succession is determined primarily by edaphic factors, especially soil pH and the availability of chemical elements, and less by the identity of dominant woody species [90]. This implies that life-form type within woody plant communities may override species identity, reinforcing the idea that species’ ecological requirements can accurately characterise the ecological structure of an ecosystem even in the absence of detailed data on species and population abundance.

The megaphanerophytes dominate the neutrophilous and neutral–basiphilous segments, and it is possible that they reflect a temporal integration of the soil pH pattern within the analysed ecosystem. Owing to their longevity and high ecological inertia, these species appear to reflect long-term soil pH conditions rather than recent fluctuations [58]. Some studies have shown that regeneration and juvenile dynamics of woody species during medium- and long-term forest restoration processes (16–30 years) are strongly influenced by local conditions and temporal context, more rapidly reflecting edaphic and microhabitat variations, including soil pH [91]. In contrast, Ellenberg indicator values for megaphanerophytes are stable and conservative, which makes these species vectors of ecological stability and continuity, thereby justifying their value for ecosystem conservation.

The pronounced variability of soil pH observed beneath different woody species, even under identical environmental conditions, confirms that soil pH is not merely a passive abiotic factor but an integrated outcome of the presence and functioning of woody species within the ecosystem. In this context, Ellenberg indicator values for soil reaction reflect not only instantaneous edaphic conditions but also the cumulative ecological imprint of woody species on the soil, being particularly relevant in mature ecosystems. Differences between life-form groups (megaphanerophytes vs. nanophanerophytes) can be explained by their different rates of temporal integration of edaphic changes, with smaller-stature species responding more rapidly to pH modifications, while long-lived species express a long-term ecological mean.

Other studies investigating the soil reaction requirements of woody species in a protected area in Central Europe have shown that long-term changes in floristic composition (over 90 years), analysed in relation to species’ requirements for the Ellenberg indicator for soil pH, indicate a decrease in soil pH as a probable driver of floristic changes. The conclusion was that slow, cumulative edaphic changes are major drivers of vegetation change, even in strictly protected areas, and that species’ requirements for the pH factor are able to capture slow, background processes (nutrients, pH), not only structural effects, being an even stronger indicator than some evident structural factors (e.g., canopy cover). These results further showed that woody species reflect dominant long-term conditions but do not prevent ecological simplification of the lower vegetation layer, being able to coexist with diversity losses in other strata; thus, a slow reorganisation of communities, driven by edaphic factors, may occur [92]. Woody species have also been referred to as “ecosystem engineers”, as they modify microclimate, soil chemistry, and resource availability, creating favourable conditions for other life forms. Changes in soil pH and C:N ratio mediated by woody species are sufficient to restructure entire biological communities, including belowground ones [93,94]. Therefore, the presence of long-lived species such as woody plants within an ecosystem, through their requirements for soil reaction, together with their influence on soil reaction (pH), can be considered key elements in defining the ecological reference framework that conditions the evolution and organisation of the rest of the phytocoenotic community.

Woody species are deeply integrated into long-term site conditions, particularly those related to soil, and they reflect dominant edaphic ecological gradients more faithfully than simple climatic variables. For example, some studies have shown that edaphic variables explain a larger proportion of vegetation structure than climate (mean annual precipitation) [95]. Accordingly, woody vegetation composition has been explained more effectively by edaphic variables than by mean annual precipitation, with soil pH and texture playing a central role in community differentiation. These findings support the interpretation of the results obtained in the analysed protected area, where the dominance of basiphilous woody species indicates long-term integration of site conditions, especially edaphic ones. Therefore, woody flora faithfully reflects dominant edaphic ecological gradients and provides a stable reference framework for ecosystem structure and functioning, representing a site history [58], which explains the stability offered by the characterisation of persistent woody life forms within an ecosystem. Ellenberg indicator values for soil pH are temporally integrated and buffered in the case of large-sized, long-lived megaphanerophytic woody species [58], functioning as integrators of the background edaphic conditions of the ecosystem.

Soil reaction analysis in the studied ecosystem demonstrates a dominance of basiphilous and neutrophilous species, with acidophilous elements being poorly represented. Nanophanerophytes prevail within the basiphilous segment, whereas megaphanerophytes dominate among neutrophilous species, suggesting vertical differentiation of ecological niches within the woody vegetation. The combined interpretation of moisture, temperature, and soil reaction values indicates that woody species composition effectively integrates multiple environmental gradients, allowing the identification of hydric, thermal, and edaphic heterogeneity within the protected ecosystem.

The ecological differentiation of plant life forms was synthesised by relating Raunkiaer bioforms to moisture, temperature, and soil reaction preferences (UTR), highlighting distinct functional patterns among mega-, meso-, and nanophanerophytes. Table 3 synthesises the ecological requirements of the studied dendroflora by relating Raunkiaer life forms to their preferences for soil moisture, temperature regime, and soil reaction (pH). The table highlights the distribution of species within each bioform across the main ecological categories, allowing a comparative assessment of functional differentiation among mega-, meso-, and nanophanerophytes. This integrative representation facilitates the identification of dominant ecological strategies associated with each life-form category and provides a concise overview of the adaptive patterns characterising the woody vegetation under study.

**Figure 10:**
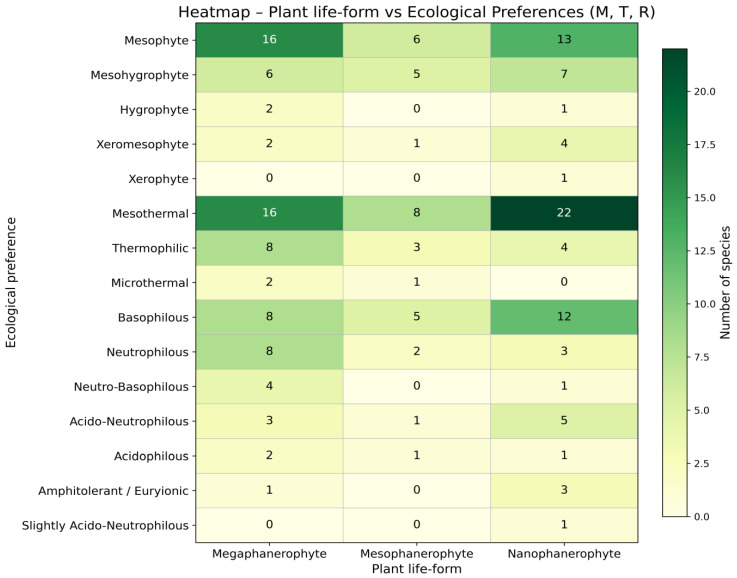
Ecological gradients inferred from Ellenberg indicator values based on woody species plant-life structure. The heatmap highlights clear gradients of moisture, temperature, and soil reaction, illustrating the role of woody flora as an integrator of long-term environmental conditions.

The correlated analysis of Ellenberg indicator values for moisture (M), temperature (T), and soil reaction (R), in relation to the structure of woody plant life forms in the analysed protected area, revealed clear ecological gradients structuring the woody flora and the presence of a predominantly mesic and mesothermal ecosystem, developed mainly on neutral to basic substrates (Figure 10).

The mesophilous segment is dominant and is equally represented by megaphanerophytes and nanophanerophytes, suggesting a vegetation structure with well-defined tree and shrub layers. The mesohygrophytic component indicates the presence of wetter microhabitats, likely associated with local edaphic conditions or topographic position, whereas xeromesophytic and xerophilous species make a limited contribution, being concentrated mainly within the nanophanerophyte category, which reflects more exposed and drier sectors. From a thermal perspective, the dominance of mesothermal species confirms the moderate temperate climatic character of the area, with a significant contribution of thermophilous elements, suggesting sub-Mediterranean influences or locally warmer conditions. Microthermic species are poorly represented and occur only within the mega- and mesophanerophyte categories, indicating a restricted segment of habitats with cooler conditions. The distribution of soil reaction highlights the predominance of basiphilous and neutrophilous species, with a reduced representation of acidophilous species, suggesting a predominantly neutral–basic substrate with moderate edaphic heterogeneity. Nanophanerophytes dominate the basiphilous segment, whereas megaphanerophytes are characteristic of the neutrophilous segment, indicating functional differentiation between vegetation layers in relation to soil chemistry. Overall, the structure of biotope factor preferences in relation to life-form type supports the existence of integrated ecological gradients, in which moisture, temperature, and soil reaction simultaneously contribute to the organisation of woody flora.

**Figure 11:**
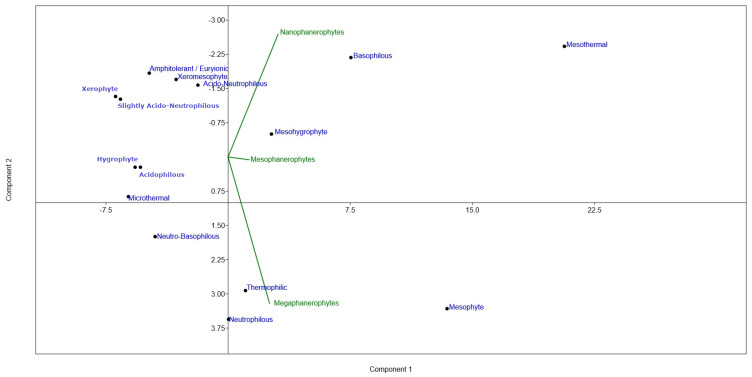
Principal Component Analysis (PCA) biplot illustrating the relationships between ecological preference categories and woody plant life forms in the studied ecosystem. Points represent ecological categories based on moisture, temperature, and soil reaction preferences, while green solid lines indicate the direction and magnitude of the contribution of each life-form category to the principal components. The distribution of categories along the axes reflects the main ecological gradients structuring the woody flora. (Component 1: Eigenvalue = 67.1338; Component 2: Eigenvalue = 4.40819).

Principal Component Analysis (PCA) revealed a clear structuring of ecological preferences (Figure 11), with the first principal component representing the dominant ecological gradient integrating moisture, temperature, and soil reaction.

The PCA biplot illustrates the relationships between ecological categories and woody life-form types. The orientation of the vectors indicates the contribution of each life-form category (megaphanerophytes, mesophanerophytes, and nanophanerophytes) to the observed ecological gradients, while the distribution of ecological categories along the principal components confirms a structured pattern. PCA highlights that life forms such as megaphanerophytes, mesophanerophytes and nanophanerophytes are not randomly distributed within the biotope, but are structured in relation to the main ecological factors. Their positioning on the biplot, in relation to the analysed ecological categories, indicates that they reflect clear differences in environmental conditions and can be interpreted as ecological indicators of the biotope. The distribution of vectors corresponding to life forms suggests the presence of well-defined ecological gradients. This positioning confirms that the analysed woody life forms characterise biotopes with a moderate level of favourability, without pronounced extremes of environmental factors. Ecological characteristics such as xerophyte, microthermal, acidophilous and hygrophyte are weakly associated with the woody life forms analysed.

Therefore, the PCA ordination supports the hypothesis of the present study that biological forms such as megaphanerophytes, mesophanerophytes and nanophanerophytes have ecological indicator value, as their presence in ecosystem is associated with the biotope gradients and is not random. Their association with specific ecological groups that reflect preferences for particular biotope conditions indicates that these life forms can be used as reference elements in the ecological characterisation of habitats.

In conclusion, the PCA indicates that the presence of the woody species in the studied ecosystem not represent merely floristic elements, but constitute indicators of ecological gradients, useful in interpreting the relationship between the biological structure of woody vegetation and environmental characteristics. These life forms generally reflect an ecological gradient of the biotope, within which mesic conditions occupy a central position and are mainly represented by megaphanerophytes and, to some extent, nanophanerophytes. This ordination confirms their indicator value in relation to environmental variation.

This distribution can be interpreted within the broader context of studies highlighting the capacity of ecological traits to reflect the long-term adaptation of woody species to environmental conditions within ecosystems. A relevant example is provided by research on woody shrubs typically classified as nanophanerophytes, which has shown that the classification of biotypes based on requirements for soil and air moisture, drought tolerance, temperature, and nutrient availability allowed the delineation of distinct climatic ecotypes and sub-ecotypes [96]. These studies demonstrate that the assignment of species to ecological categories such as mesophytes, mesohygrophytes, mesoxerophytes, or xeromesophytes does not represent merely a static description, but rather reflects real functional differences in ecological plasticity and in the capacity of species to integrate climatic variability.

Furthermore, changes in the structure of woody flora and its associated components can be used as indicators of environmental change, even in the absence of continuous direct measurements of abiotic factors, solely by establishing species’ ecological preferences for biotope factors through Ellenberg indicator values. A study analysing mature spruce forests dominated by Picea abies [97] showed that these forests undergo significant structural and functional changes as a result of increasing temperatures and intensified drought episodes, and that these changes can be detected through modifications occurring in the lower tree layer following the degradation of the upper canopy layer. Such changes include, for example, an increased proportion of thermophilous and basiphilous species, indicating a clear transition from a boreal to a nemoral spectrum, and reflecting a climatic and edaphic gradient induced by climate change. It follows that the lower layers of vegetation and the dynamics of ecological succession can undergo profound changes that are detectable through the analysis of the ecological requirements of floristic communities, as determined using Ellenberg scales.

A comparable approach has been applied in Mediterranean lowland oak floodplain forests, where long-term re-survey analyses integrating life forms, chorotypes and Ellenberg indicator values revealed species turnover without substantial changes in the overall bioindicative structure of the ecosystem. Despite shifts in floristic composition following drought-induced tree dieback, the ecological signal inferred from EIV remained relatively stable, suggesting ecosystem resilience. Similarly, in the present study, the dominance of mesophilous and mesothermic woody species—largely represented by megaphanerophytes and nanophanerophytes—indicates a stable ecological framework. The long lifespan and structural persistence of woody plants allow them to integrate environmental conditions over extended temporal scales, thereby reflecting the fundamental climatic and edaphic setting of the protected area rather than short-term fluctuations. These findings support the reliability of Ellenberg-based analyses in detecting both long-term stability and directional change in forest ecosystems [98]. Although mesophilous and mesothermal species dominate the woody flora of the protected area, these ecological categories are represented mainly by megaphanerophytes and nanophanerophytes, rather than by mesophanerophytes. This pattern does not indicate a structural inconsistency, but rather reflects the fact that ecological preferences (mesophily and mesothermy) describe requirements related to moisture and temperature regimes, whereas life forms express the structural position of species within the vegetation. The prevalence of megaphanerophytes within the mesophilous–mesothermal segment is consistent with the dominance of forest species adapted to moderate climatic conditions. At the same time, the proportion of nanophanerophytes within the same category suggests an important contribution of small-stature woody forms to the structure of the analysed flora, without necessarily implying a quantitative interpretation of vertical stratification. The lower proportion of mesophanerophytes can be explained by the intermediate character of this life-form category. Overall, the association between mesophilous–mesothermal dominance and the prevalence of persistent woody life forms indicates the long-term integration of dominant climatic and edaphic conditions. Some studies provide results indicating a relative stability of understory diversity in mixed forests following drought episodes [99], suggesting that vertical structure and the composition of the upper layer can modulate ecosystem responses to climatic stress. In this context, the dominance of megaphanerophytes and nanophanerophytes within the mesophilous-mesothermal segment identified in the analysed protected area reflects a structural organisation characteristic of persistent forest ecosystems. The prevalence of these life forms, associated with the longevity of woody species, provides the premise for a system capable of integrating dominant climatic and edaphic conditions over the long term and of maintaining the functional coherence of the ecosystem. It is precisely this capacity to reflect and conserve the fundamental ecological framework that confers a major diagnostic value on woody flora. Protecting the studied area therefore means protecting a system in which biological structure and environmental conditions are in a historically consolidated balance, and maintaining this balance becomes essential in the context of intensifying climate change. The structure of woody species reflects dominant long-term environmental conditions, and vegetation responses are closely dependent on habitat type, following different pathways according to the biotope. These species not only reflect environmental conditions but also integrate and signal long-term ecological strategies, being sensitive to large-scale climatic changes. In this context, woody species can indicate future processes of pronounced decline, constituting relevant tools for assessing ecosystem condition and trajectories [97]. A framework based on multiple ecological indicators allows the identification of vulnerable habitats and provides relevant information for conservation and management, even in complex contexts, thereby justifying the use of woody flora as an ecological assessment tool in ecosystems [25].

In the context of the above considerations, the results of the present study, obtained through the use of Ellenberg ecological indicator values, show that species structure and life-form structure faithfully reflect the dominant gradients of moisture and temperature. The dominance of mesophilous and mesothermal elements, integrated mainly by long-lived woody life forms, supports the idea that persistent vegetation provides a synthetic representation of long-term environmental conditions. It follows that, in both herbaceous and forest ecosystems, floristic structure and the distribution of ecological groups constitute robust indicators of ecosystem functioning, anthropogenic pressures, and ecological stability, providing a solid basis for interpreting environmental gradients and for supporting conservation decisions. Similarly, in the case of the woody flora analysed in the studied protected area, the analysis of vegetation structure through ecological indicators of biotope requirements provides a sound basis for evaluating ecosystem stability and integrity.

The results obtained in the present study, based on the distribution of life forms in relation to Ellenberg ecological requirements, fit the pattern described by recent studies regarding the decoupling of responses between herbaceous and woody plants along ecological gradients. The dominance of mesophilous and mesothermal categories in the analysed woody flora, together with the prevalence of megaphanerophytes and nanophanerophytes within these ecological groups, indicates that community structure is primarily controlled by a background ecological filter associated with long-term climatic and edaphic conditions. The Ellenberg values used therefore appear to be ecologically relevant and not distorted by opportunistic species. The distribution of ecological requirements of woody species thus reflects not only current environmental conditions but also the degree of ecosystem resilience, confirming the usefulness of Ellenberg analysis combined with life-form type as a tool for assessing conservation status and vulnerability of forest ecosystems. The analysis of Ellenberg ecological indices and life-form structure highlights the presence of well-defined ecological gradients within the national park, from hydric, thermal, and edaphic perspectives. The concurrent presence of xerophilous, mesophilous, and hygrophilous species, as well as microthermic, mesothermal, and thermophilous taxa, reflects the heterogeneity of environmental conditions and habitat diversity, characteristics typical of a protected area with high conservation value.

## 5. Conclusions

The ecological structure of the woody flora reflects the ecological gradients of the studied ecosystem.

The dominance of mesophilous and mesothermal woody species, particularly within megaphanerophytes and nanophanerophytes, indicates that woody vegetation reliably reflects the main hydric, thermal, and edaphic gradients of the ecosystem. The high proportion of native species and the limited presence of alien taxa suggest a well-preserved ecosystem with high ecological integrity and resistance to biological invasions.

Overall, the results indicate that woody species can serve as valuable structural and ecological indicators, providing insights into the ecological gradients of ecosystems.

These findings support the use of woody life-form structure combined with ecological indicator values as a robust approach for interpreting ecological gradients and ecosystem stability in protected temperate areas. However, this approach should be regarded as complementary and not as a substitute for comprehensive vegetation analyses.

## Figures and Tables

**Table 1:** Taxonomic composition, life forms, phytogeographical affiliation, and biogeographical status of the woody plant species.

No.	Species	Family	Plant Life-Form	Phytogeographic Element	Biogeographical Status
1	*Taxus baccata* L.	Taxaceae	Mesophanerophyte	European	N
2	*Abies alba* Mill.	Pinaceae	Megaphanerophyte	European	N
3	*Picea abies* (L.) H.Karst.	Pinaceae	Megaphanerophyte	Circumboreal	N
4	*Pinus nigra* J.F.Arnold	Pinaceae	Megaphanerophyte	European	N
5	*Carpinus betulus* L.	Betulaceae	Megaphanerophyte	European	N
6	*Carpinus orientalis* Mill.	Betulaceae	Mesophanerophyte	Balkan–Submediterranean	N
7	*Corylus colurna* L.	Betulaceae	Megaphanerophyte	Balkan–Anatolian	N
8	*Corylus avellana* L.	Betulaceae	Mesophanerophyte	European	N
9	*Alnus glutinosa* (L.) Gaertn.	Betulaceae	Megaphanerophyte	Eurasian	N
10	*Juglans regia* L.	Juglandaceae	Megaphanerophyte	Irano-Turanian	AH
11	*Populus alba* L.	Salicaceae	Megaphanerophyte	Eurasian	N
12	*Populus nigra* L.	Salicaceae	Megaphanerophyte	Eurasian	N
13	*Salix alba* L.	Salicaceae	Megaphanerophyte	Eurasian	N
14	*Salix purpurea* L.	Salicaceae	Nanophanerophyte	Eurasian	N
15	*Salix caprea* L.	Salicaceae	Mesophanerophyte	European	N
16	*Morus alba* L.	Moraceae	Megaphanerophyte	East Asian	NN
17	*Quercus cerris* L.	Fagaceae	Megaphanerophyte	European	N
18	*Quercus petraea* (Matt.) Liebl.	Fagaceae	Megaphanerophyte	European	N
19	*Quercus frainetto* Ten.	Fagaceae	Megaphanerophyte	Balkan	N
20	*Quercus pubescens* Willd.	Fagaceae	Megaphanerophyte	European	N
21	*Fagus sylvatica* L.	Fagaceae	Megaphanerophyte	European	N
22	*Ulmus minor* Mill.	Ulmaceae	Megaphanerophyte	European	N
23	*Ulmus procera* Salisb.	Ulmaceae	Megaphanerophyte	European	NN
24	*Viscum album* L.	Santalaceae	Nanophanerophyte	Eurasian	N
25	*Clematis vitalba* L.	Ranunculaceae	Mesophanerophyte	Submediterranean	N
26	*Helianthemum canum* (L.) Baumg.	Cistaceae	Nanophanerophyte	European	N
27	*Helianthemum nummularium* (L.) Mill.	Cistaceae	Nanophanerophyte	European	N
28	*Tilia tomentosa* Moench	Malvaceae (s.l.)	Megaphanerophyte	Balkan	N
29	*Tilia cordata* Mill.	Malvaceae (s.l.)	Megaphanerophyte	European	N
30	*Tilia platyphyllos* Scop.	Malvaceae (s.l.)	Megaphanerophyte	European	N
31	*Acer platanoides* L.	Sapindaceae	Megaphanerophyte	European	N
32	*Acer campestre* subsp. *marsicum* (Guss.) K. Koch	Sapindaceae	Mesophanerophyte	European	N
33	*Acer pseudoplatanus* L.	Sapindaceae	Megaphanerophyte	European	N
34	*Acer tataricum* L.	Sapindaceae	Mesophanerophyte	Pontic–Pannonian	N
35	*Cornus mas* L.	Cornaceae	Nanophanerophyte	Submediterranean	N
36	*Cornus sanguinea* L.	Cornaceae	Nanophanerophyte	European	N
37	*Spiraea ulmifolia* Scop.	Rosaceae	Nanophanerophyte	European	N
38	*Cotoneaster tomentosus* (Aiton) Lindl.	Rosaceae	Nanophanerophyte	European	N
39	*Cotoneaster integerrimus* Medik.	Rosaceae	Nanophanerophyte	European	N
40	*Malus sylvestris* (L.) Mill.	Rosaceae	Mesophanerophyte	European	N
41	*Sorbus aucuparia* L.	Rosaceae	Mesophanerophyte	Circumboreal	N
42	*Sorbus domestica* L.	Rosaceae	Megaphanerophyte	Submediterranean	N
43	*Crataegus monogyna* Jacq.	Rosaceae	Nanophanerophyte	European	N
44	*Rubus idaeus* L.	Rosaceae	Nanophanerophyte	Circumboreal	N
45	*Rubus sulcatus* Vest	Rosaceae	Nanophanerophyte	European	N
46	*Rubus banaticus* Nyar.	Rosaceae	Nanophanerophyte	Carpathian endemic	N
47	*Rubus tomentosus* Borkh.	Rosaceae	Nanophanerophyte	European	N
48	*Rubus hirtus* Waldst. & Kit.	Rosaceae	Nanophanerophyte	European	N
49	*Rubus caesius* L.	Rosaceae	Nanophanerophyte	Eurasian	N
50	*Rosa canina* L.	Rosaceae	Nanophanerophyte	European	N
51	*Rosa pendulina* L.	Rosaceae	Nanophanerophyte	European	N
52	*Prunus spinosa* L.	Rosaceae	Nanophanerophyte	European	N
53	*Cotinus coggygria* Scop.	Anacardiaceae	Nanophanerophyte	Submediterranean	N
54	*Genista tinctoria* L.	Fabaceae	Nanophanerophyte	European	N
55	*Genista sagittalis* L.	Fabaceae	Nanophanerophyte	European	N
56	*Hedera helix* L.	Araliaceae	Mesophanerophyte	Eurasian	N
57	*Vinca minor* L.	Apocynaceae	Nanophanerophyte	Submediterranean	N
58	*Fraxinus ornus* L.	Oleaceae	Megaphanerophyte	Submediterranean	N
59	*Fraxinus excelsior* L.	Oleaceae	Megaphanerophyte	European	N
60	*Syringa vulgaris* L.	Oleaceae	Mesophanerophyte	Balkan	AH
61	*Ligustrum vulgare* L.	Oleaceae	Nanophanerophyte	Submediterranean	N
62	*Sambucus nigra* L.	Adoxaceae	Mesophanerophyte	European	N
63	*Viburnum lantana* L.	Adoxaceae	Nanophanerophyte	Submediterranean	N
64	*Lonicera xylosteum* L.	Caprifoliaceae	Nanophanerophyte	European	N

N = Native species; NN = Non-native species; AH = Archaeophyte species.

**Table 2:** Taxonomic composition and ecological preferences for moisture, temperature and soil (pH) reaction of the woody plant species.

No.	Species	Family	Ecological Preferences		
			M (Moisture)	T (Temperature)	R (Soil Reaction)
1	*Taxus baccata* L.	Taxaceae	Mesohygrophyte	Mesothermal	Neutro-Basophilous
2	*Abies alba* Mill.	Pinaceae	Mesohygrophyte	Microthermal	Acido-Neutrophilous
3	*Picea abies* (L.) H.Karst.	Pinaceae	Mesohygrophyte	Microthermal	Acidophilous
4	*Pinus nigra* J.F.Arnold	Pinaceae	Mesophyte	Mesothermal	Basophilous
5	*Carpinus betulus* L.	Betulaceae	Mesophyte	Mesothermal	Neutrophilous
6	*Carpinus orientalis* Mill.	Betulaceae	Xeromesophyte	Thermophilic	Basophilous
7	*Corylus colurna* L.	Betulaceae	Mesophyte	Mesothermal	Basophilous
8	*Corylus avellana* L.	Betulaceae	Mesophyte	Mesothermal	Amphitolerant (Euryionic)
9	*Alnus glutinosa* (L.) Gaertn.	Betulaceae	Hygrophyte	Mesothermal	Neutrophilous
10	*Juglans regia* L.	Juglandaceae	Mesophyte	Thermophilic	Neutro-Basophilous
11	*Populus alba* L.	Salicaceae	Mesohygrophyte	Mesothermal	Basophilous
12	*Populus nigra* L.	Salicaceae	Mesohygrophyte	Mesothermal	Neutro-Basophilous
13	*Salix alba* L.	Salicaceae	Hygrophyte	Mesothermal	Neutrophilous
14	*Salix purpurea* L.	Salicaceae	Hygrophyte	Mesothermal	Neutrophilous
15	*Salix caprea* L.	Salicaceae	Mesohygrophyte	Mesothermal	Acido-Neutrophilous
16	*Morus alba* L.	Moraceae	Mesophyte	Thermophilic	Amphitolerant (Euryionic)
17	*Quercus cerris* L.	Fagaceae	Mesophyte	Mesothermal	Acido-Neutrophilous
18	*Quercus petraea* (Matt.) Liebl.	Fagaceae	Mesophyte	Mesothermal	Acidophilous
19	*Quercus frainetto* Ten.	Fagaceae	Mesophyte	Thermophilic	Neutro-Basophilous
20	*Quercus pubescens* Willd.	Fagaceae	Xeromesophyte	Thermophilic	Basophilous
21	*Fagus sylvatica* L.	Fagaceae	Mesohygrophyte	Mesothermal	Acido-Neutrophilous
22	*Ulmus minor* Mill.	Ulmaceae	Mesophyte	Mesothermal	Neutrophilous
23	*Ulmus procera* Salisb.	Ulmaceae	Mesophyte	Mesothermal	Neutrophilous
24	*Viscum album* L.	Santalaceae	Mesophyte	Mesothermal	Amphitolerant (Euryionic)
25	*Clematis vitalba* L.	Ranunculaceae	Mesophyte	Mesothermal	Basophilous
26	*Helianthemum canum* (L.) Baumg.	Cistaceae	Xerophyte	Thermophilic	Basophilous
27	*Helianthemum nummularium* (L.) Mill.	Cistaceae	Xeromesophyte	Mesothermal	Basophilous
28	*Tilia tomentosa* Moench	Malvaceae (s.l.)	Mesophyte	Mesothermal	Neutrophilous
29	*Tilia cordata* Mill.	Malvaceae (s.l.)	Xeromesophyte	Thermophilic	Basophilous
30	*Tilia platyphyllos* Scop.	Malvaceae (s.l.)	Mesophyte	Mesothermal	Neutro-Basophilous
31	*Acer platanoides* L.	Sapindaceae	Mesophyte	Mesothermal	Neutrophilous
32	*Acer campestre* subsp. *marsicum* (Guss.) K. Koch	Sapindaceae	Mesohygrophyte	Microthermal	Acidophilous
33	*Acer pseudoplatanus* L.	Sapindaceae	Mesophyte	Thermophilic	Basophilous
34	*Acer tataricum* L.	Sapindaceae	Mesophyte	Mesothermal	Basophilous
35	*Cornus mas* L.	Cornaceae	Mesophyte	Thermophilic	Neutro-Basophilous
36	*Cornus sanguinea* L.	Cornaceae	Mesohygrophyte	Mesothermal	Neutrophilous
37	*Spiraea ulmifolia* Scop.	Rosaceae	Mesohygrophyte	Mesothermal	Basophilous
38	*Cotoneaster tomentosus* (Aiton) Lindl.	Rosaceae	Mesophyte	Mesothermal	Neutrophilous
39	*Cotoneaster integerrimus* Medik.	Rosaceae	Mesophyte	Thermophilic	Basophilous
40	*Malus sylvestris* (L.) Mill.	Rosaceae	Mesohygrophyte	Mesothermal	Neutrophilous
41	*Sorbus aucuparia* L.	Rosaceae	Mesophyte	Thermophilic	Basophilous
42	*Sorbus domestica* L.	Rosaceae	Mesophyte	Thermophilic	Basophilous
43	*Crataegus monogyna* Jacq.	Rosaceae	Mesophyte	Mesothermal	Amphitolerant (Euryionic)
44	*Rubus idaeus* L.	Rosaceae	Mesohygrophyte	Mesothermal	Acido-Neutrophilous
45	*Rubus sulcatus* Vest	Rosaceae	Mesohygrophyte	Mesothermal	Acido-Neutrophilous
46	*Rubus banaticus* Nyar.	Rosaceae	Mesohygrophyte	Mesothermal	Acido-Neutrophilous
47	*Rubus tomentosus* Borkh.	Rosaceae	Mesohygrophyte	Mesothermal	Acido-Neutrophilous
48	*Rubus hirtus* Waldst. & Kit.	Rosaceae	Mesohygrophyte	Mesothermal	Acido-Neutrophilous
49	*Rubus caesius* L.	Rosaceae	Mesophyte	Mesothermal	Amphitolerant (Euryionic)
50	*Rosa canina* L.	Rosaceae	Mesophyte	Mesothermal	Basophilous
51	*Rosa pendulina* L.	Rosaceae	Mesophyte	Mesothermal	Basophilous
52	*Prunus spinosa* L.	Rosaceae	Mesophyte	Mesothermal	Basophilous
53	*Cotinus coggygria* Scop.	Anacardiaceae	Xeromesophyte	Thermophilic	Basophilous
54	*Genista tinctoria* L.	Fabaceae	Xeromesophyte	Mesothermal	Slightly Acido-Neutrophilous
55	*Genista sagittalis* L.	Fabaceae	Xeromesophyte	Mesothermal	Acidophilous
56	*Hedera helix* L.	Araliaceae	Mesophyte	Mesothermal	Amphitolerant (Euryionic)
57	*Vinca minor* L.	Apocynaceae	Mesophyte	Mesothermal	Basophilous
58	*Fraxinus ornus* L.	Oleaceae	Mesophyte	Thermophilic	Basophilous
59	*Fraxinus excelsior* L.	Oleaceae	Mesohygrophyte	Mesothermal	Neutrophilous
60	*Syringa vulgaris* L.	Oleaceae	Mesophyte	Thermophilic	Basophilous
61	*Ligustrum vulgare* L.	Oleaceae	Mesophyte	Mesothermal	Basophilous
62	*Sambucus nigra* L.	Adoxaceae	Mesohygrophyte	Mesothermal	Amphitolerant (Euryionic)
63	*Viburnum lantana* L.	Adoxaceae	Mesophyte	Mesothermal	Basophilous
64	*Lonicera xylosteum* L.	Caprifoliaceae	Mesophyte	Mesothermal	Basophilous

**Table 3:** Distribution of ecological preferrences (moisture–temperature–soil reaction) across woody plant life-forms.

Plant Life-Form	Moisture (M) Requirements	Number of Species	Temperature (T) Requirements	Number of Species	Soil Reaction (R) Requirements	Number of Species
**Megaphanerophyte** (*n* = 26)	Mesophyte	16	Mesothermal	16	Basophilous	8
	Mesohygrophyte	6	Thermophilic	8	Neutrophilous	8
	Hygrophyte	2	Microthermal	2	Neutro-Basophilous	4
	Xeromesophyte	2			Acido-Neutrophilous	3
					Acidophilous	2
					Amphitolerant (Euryionic)	1
**Mesophanerophyte** (*n* = 12)	Mesophyte	6	Mesothermal	8	Amphitolerant (Euryionic)	0
	Mesohygrophyte	5	Thermophilic	3	Basophilous	5
	Xeromesophyte	0	Microthermal	1	Neutrophilous	2
					Acido-Neutrophilous	1
					Acidophilous	1
**Nanophanerophyte** (*n* = 26)	Mesophyte	13	Mesothermal	22	Basophilous	12
	Mesohygrophyte	7	Thermophilic	4	Acido-Neutrophilous	5
	Xeromesophyte	4			Neutrophilous	3
					Neutro-Basophilous	1
	Xerophyte	1			Amphitolerant (Euryionic)	3
	Hygrophyte	1			Acidophilous	1
					Slightly Acido-Neutrophilous	1

## Data Availability

The original contributions presented in this study are included in the article/supplementary material. Further inquiries can be directed to the corresponding author.

## Supplementary Materials

The following supporting information can be downloaded at: https://www.mdpi.com/article/10.3390/plants15081194/s1.
